# Disease Isolates of *Streptococcus pseudopneumoniae* and Non-Typeable *S. pneumoniae* Presumptively Identified as Atypical *S. pneumoniae* in Spain

**DOI:** 10.1371/journal.pone.0057047

**Published:** 2013-02-21

**Authors:** Dora Rolo, Alexandra S. Simões, Arnau Domenech, Asunción Fenoll, Josefina Liñares, Hermínia de Lencastre, Carmen Ardanuy, Raquel Sá-Leão

**Affiliations:** 1 Institut d'Investigació Biomèdica de Bellvitge, Hospital Universitari de Bellvitge, Microbiology Department, Universistat de Barcelona, Barcelona, Spain; 2 Centro de investigación en red de enfermedades respiratorias, Instituto de Salud Carlos III, Madrid, Spain; 3 Laboratory of Molecular Genetics, Instituto de Tecnologia Química e Biológica, Universidade Nova de Lisboa, Oeiras, Portugal; 4 Laboratory of Molecular Microbiology of Human Pathogens, Instituto de Tecnologia Química e Biológica, Universidade Nova de Lisboa, Oeiras, Portugal; 5 National Center for Microbiology, Instituto de Salud Carlos III, Madrid, Spain; 6 Laboratory of Microbiology, The Rockefeller University, New York, New York, United States of America; Instituto Butantan, Brazil

## Abstract

We aimed to obtain insights on the nature of a collection of isolates presumptively identified as atypical *Streptococcus pneumoniae* recovered from invasive and non-invasive infections in Spain. One-hundred and thirty-two isolates were characterized by: optochin susceptibility in ambient and CO_2_-enriched atmosphere; bile solubility; PCR-based assays targeting pneumococcal genes *lyt*A, *ply*, *psp*A, *cps*A, *Spn*9802, *ali*B-like ORF2, and a specific 16S rRNA region; multilocus sequence analysis; and antimicrobial susceptibility. By multilocus sequence analysis, 61 isolates were *S. pseudopneumoniae*, 34 were pneumococci, 13 were *S. mitis*, and 24 remained unclassified as non-pneumococci. Among *S. pseudopneumoniae* isolates, 51 (83.6%) were collected from respiratory tract samples; eight isolates were obtained from sterile sources. High frequency of non-susceptibility to penicillin (60.7%) and erythromycin (42.6%) was found. Only 50.8% of the *S. pseudopneumoniae* isolates displayed the typical optochin phenotype originally described for this species. None harbored the *cps*A gene or the pneumococcal typical *lyt*A restriction fragment length polymorphism. The *Spn*9802 and the specific 16S rRNA regions were detected among the majority of the *S. pseudopneumoniae* isolates (n = 59 and n = 49, respectively). The *ply* and *psp*A genes were rarely found. A high genetic diversity was found and 59 profiles were identified. Among the *S. pneumoniae*, 23 were capsulated and 11 were non-typeable. Three non-typeable isolates, associated to international non-capsulated lineages, were recovered from invasive disease sources. In conclusion, half of the atypical pneumococcal clinical isolates were, in fact, *S. pseudopneumoniae* and one-fourth were other streptococci. We identified *S. pseudopneumoniae* and non-typeable pneumococci as cause of disease in Spain including invasive disease.

## Introduction

*Streptococcus pneumoniae* (pneumococcus) is an important human pathogen worldwide responsible for systemic diseases such as meningitis, pneumonia, and bacteraemia. [1], [2] Culture-based identification methods usually rely on colony morphology, optochin susceptibility, bile solubility, and agglutination by the Quellung reaction. [3] However, exceptions have been described and include pneumococci that are optochin-resistant, [4], [5] bile-insoluble, [6] and do not have a specific agglutination in the Quellung reaction due to lack of capsule. [7], [8] This latter group is generally called non-typeable pneumococci and is often found in colonization. [7], [9] Although sporadically, non-typeable pneumococci have also been associated with disease such as conjunctivitis (including large outbreaks), [10], [11] acute otitis media, [12] acute exacerbations in patients with chronic obstructive pulmonary disease (COPD), [13] and more recently in invasive disease. [14].

Pneumococcal isolates displaying odd properties in the assays described above have been collectively named atypical pneumococci and are often difficult to identify. On the other hand, sporadic isolates of closely-related species that have one or more properties typically associated with pneumococci have been described. [9], [15], [16].

In 2004, Arbique and colleagues identified some of these atypical pneumococci as a new species – *Streptococcus pseudopneumoniae.* [17] Although similar to pneumococci, they were characterized by being bile insoluble and optochin-resistant when incubated under a 5% CO_2_ atmosphere but optochin–susceptible when incubated under ambient atmosphere. *S. pseudopneumoniae* have been identified among colonizing children and respiratory samples. [15], [18] Although, their clinical relevance has not been clearly established, *S. pseudopneumoniae* have been associated with COPD, [19] and its disease potential has been demonstrated in mice models of peritonitis and sepsis. [20].

As biochemical tests are often insufficient to distinguish atypical *S. pneumonia*e from *S. pseudopneumoniae* or other closely related streptococci several molecular assays have been proposed. The construction of phylogenetic trees using six concatenated multilocus sequence typing (MLST) alleles, called Multilocus Sequence Analysis (MLSA), is considered a good approach to differentiate *S. pneumoniae* from closely related species. [15], [21] In addition, several other assays have been developed most of which are PCR-based and target specific pneumococcal virulence factors, such as autolysin A (*lyt*A), pneumolysin (*ply*), pneumococcal surface protein A (*psp*A), or the capsular polysaccharide biosynthesis gene A (*cps*A). [6], [15] Unknown putative genes, specific intergenic DNA sequences, or specific regions of the 16S rRNA, have also been proposed to be pneumococcal species-specific. [22], [23] However, the occurrence of *Streptococcus mitis* isolates harbouring genes encoding *S. pneumoniae* virulence factors has been reported and whether the genetic assays recently proposed universally distinguish pneumococci from the closely related species remains to be seen. [15], [24], [25], [26], [27].

In this study, we aimed to characterize a large collection of invasive and non-invasive disease isolates obtained in Spain, which had been identified as atypical pneumococci. We have combined MLSA with a panel of phenotypic and molecular assays in order to gain insights on the nature of such isolates.

## Materials and Methods

### Ethics Statement

This study and publication of the results were approved by the “Comité Ètic d'Investigació Clínica del Hospital Universitari de Bellvitge” and written or oral informed consent was considered not necessary, because data were analyzed anonymously.

### Bacterial Isolates

A total of 132 clinical isolates classified as non-(sero)typeable or atypical pneumococci collected at two Spanish laboratories were included in the study. There were no duplicates within or between the two sets studied.

The first set comprised 56 isolates collected at the Spanish Reference Pneumococcal Laboratory (Centro Nacional de Microbiologia, ISCIII, Madrid, Spain), which receives pneumococcal disease isolates from 190 hospitals throughout the entire country. The isolates were obtained between 2004 and 2009, and were mostly (44 out of 56) from non-sterile sites. This set represented 7.7% (56 out of 728) of the total non-(sero)typeable or atypical pneumococci *S. pneumoniae* isolated during that period which, in turn, corresponded to 4.6% of all pneumococcal isolates identified in the same period. This set included: i) 44 specimens with atypical pneumococcal identification [optochin resistant in CO_2_ atmosphere, bile negative, and Accuprobe™ positive (Gen-Probe, San Diego, California)] of which 43 had been isolated from non-sterile sites; and ii) 12 non-typeable pneumococci (optochin susceptible in CO_2_ atmosphere, and showing no agglutination in the Quellung reaction), of which eight were invasive isolates.

The second set comprised 76 isolates collected at the tertiary adult Hospital Universitari de Bellvitge (Barcelona, Spain) obtained between 1991 and 2009 and were mostly (63 out of 76) from non-sterile sites. This set represented 43.9% (76 out of 173) of the total non-(sero)typeable or atypical pneumococci *S. pneumoniae* isolated during that period which, in turn, corresponded to 5.1% of all pneumococcal isolates identified in the same period. This collection also include two groups of isolates: i) 35 specimens with atypical pneumococcal identification [reduced optochin susceptibility in CO_2_ atmosphere, positive Slidex® pneumo-Kit aglutination test (bioMérieux, Marcy-l’Etoile, France)] of which 30 had been isolated from non-sterile sites; and ii) 41 non-typeable pneumococci (optochin susceptible in CO_2_ atmosphere and showing no agglutination in the Quellung reaction), of which eight were invasive isolates.

In the total collection invasive isolates were obtained from blood (n = 11), bronchoalveolar lavage (n = 7), transthoracic needle aspiration (n = 1), cerebrospinal fluid (n = 1), bronchoscopic-protected catheter brush (n = 1) and ascitic fluid (n = 1). Non-invasive isolates were obtained from sputum (n = 75), bronchial aspiration (n = 23), conjunctiva swab (n = 4), and others (n = 8).

### Optochin Susceptibility

Optochin susceptibility was tested by disk diffusion, using commercially available optochin disks (5 µg; 6 mm; Oxoid, Hampshire, England) applied onto blood agar plates (trypticase soy agar supplemented with 5% sheep blood), which had been inoculated with a 0.5 McFarland standard suspension of the culture to be tested. Plates (two per isolate) were incubated in parallel overnight at 37°C in a 5% CO_2_ and ambient atmosphere as described by Arbique *et al*. to differentiate *S. pneumoniae* from *S. pseudopneumoniae.* [*17*] Isolates were considered to be resistant to optochin if they displayed inhibition zones smaller than 14 mm. [17].

### Bile Solubility Test

The bile solubility assay was performed according to standard procedures described by Rouff *et al*. [3].

### Antimicrobial Susceptibility Testing

Antimicrobial susceptibility against penicillin, cefotaxime, erythromycin, clindamycin, cotrimoxazole, tetracycline, ciprofloxacin, levofloxacin and chloramphenicol was performed by disk-diffusion and microdilution method, following the recommendations and definitions of the Clinical and Laboratory Standards Institute (CLSI). [28] In particular, for penicillin, pneumococcal oral penicillin V breakpoints were used (S:≤0.06, I:0.12-1, R:≥2); for cefotaxime, pneumococcal meningeal breakpoints were used (S:≤0.5, I:1, R:≥2). For ciprofloxacin, an MIC≥4 mg/L was considered resistant.

### Capsular Typing

For pneumocccal capsular detection, isolates were serotyped by the Quellung reaction, and/or by a PCR-based assay following the protocols described by the CDC. [29], [30] Isolates for which a capsule could not be assigned were probed against Omniserum (Statens Serum Institute, Copenhagen, Denmark), a serum that contains antibodies to all known pneumococcal types.

### Multiplex PCR for Detection of *lytA*, *cpsA* and *ali*B-like ORF2

A multiplex PCR assay was used to distinguish *S. pneumoniae* from closely related species as previously described. [9] This multiplex PCR detects internal fragments of *cps*A (a conserved pneumococcal capsular polysaccharide gene); *lytA* (the major pneumococcal autolysin); *ali*B-like ORF2 (a gene described as frequently present in the capsular region of non-capsulated pneumococci); [8] and 16S rRNA (positive internal control).

### PCR Screening for Additional Putative Specific Pneumococcal Signatures - *psp*A, *Spn*9802 and *16S* rRNA

Screening for the presence of *pspA* (the gene that encodes for the pneumococcal surface protein A), *Spn*9802 (a genetic region which encodes for a protein of unknown function that has initially been described as a specific target for *S. pneumoniae*), and a 16S rRNA allele that has been described as pneumococcal-specific, was done as described. [22], [23], [31].

### *lytA* RFLP Signatures

The *lytA* gene was amplified by PCR and RFLP signatures characteristic of typical pneumococcal *lytA* or atypical (non-pneumococcal) *lytA* were determined by digesting the amplification product with BsaAI and separating the fragments by agarose gel electrophoresis, as published. [16].

### *ply* and *mly* PCR Detection and RFLP Signatures

The presence of *ply* (encoding pneumolysin, a cholesterol-dependent pneumococcal citolysin) or *mly* (a *ply* homologue identified in some *S. mitis* isolates), [32] was screened by digesting the amplification product with BsaAI and separating the fragments by agarose gel electrophoresis, as published. [15].

### Multilocus Sequence Typing (MLST)

The amplification of internal fragments of seven housekeeping genes (*aroE*, *gdh*, *gk*i, *recP*, *spi*, *xpt*, and *ddl*) and allele assignment were carried out essentially as described in the international pneumococcal MLST database. [33] Sequencing was performed at Macrogen, Inc. (Seoul, Korea) and the sequencing analysis was conducted with DNAStar (Lasergene). For non-pneumococcal isolates allele assignment was done internally using arbitrary numbers following the same principles of the published MLST schemes. The eBURST algorithm [34] was used for determining the population structure of the *S. pseudopneumoniae* isolates. Two strains were considered in the same clonal complex when at least four of the six alleles were identical (the *ddl* allele was not systematically determined for these isolates and was thus excluded from the analysis). Nucleotide sequences were submitted to the GenBank database (submission grp 3980184) and are also available from the corresponding author.

### Multilocus Sequence Analysis (MLSA)

Phylogenetic analysis using MLST data was done by concatenating the sequences of all MLST loci except *ddl* to obtain one single sequence of 2,758 bp. [21] MLST allele sequences of *S. pneumoniae, S. mitis*, *S. pseudopneumoniae*, and *S. oralis* previously described were used as controls. [15], [35], [36], [37] Phylogenetic and molecular evolutionary analyses were conducted using MEGA version 4 as previously described. [15], [38].

## Results and Discussion

To obtain insights on the nature and characteristics of 132 Spanish isolates presumptively identified as atypical pneumococci recovered from invasive and non-invasive disease sources, we performed several phenotypic and genotypic assays.

**Figure 1:**
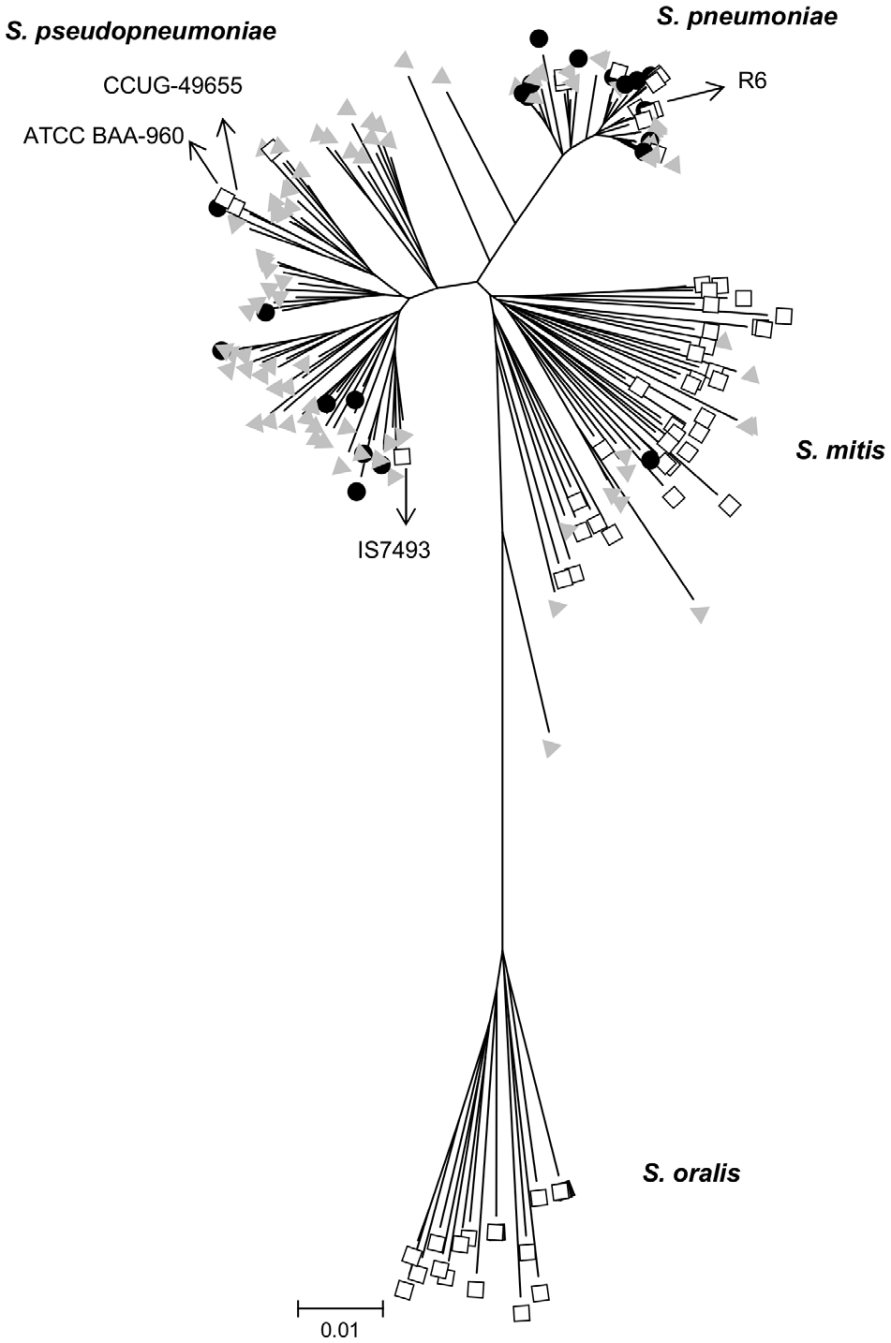
Genetic relationships of the strains determined by MLSA. The symbols indicate: grey triangle, non-invasive disease strains; black circle, invasive disease strains; white square, strains described in other studies. [15], [17], [35], [36], [37].

For species assignment MLSA was performed as described previously using the study isolates as well as the collections previously described by Chi et al. and Simões et al. [15], [35] For 22 isolates one or more MLST alleles could not be obtained despite repeated attempts using various primers and several different amplification conditions. For this reason, these isolates were not fully characterized. For the 110 remaining isolates MLSA was performed and identified 61 isolates as *S. pseudopneumoniae*, 34 as *S. pneumoniae,* and 13 as *S. mitis*; within the *S. pneumoniae* branch two outliers closer to the root of the tree were noted and these remained unidentified (Figure 1). Isolates which are clearly closely related to *S. pneumoniae* but for which species assignment is not obvious have also been described by others. [39].

Overall, the 22 invasive isolates were identified as 12 *S. pneumoniae*, 8 *S. pseudopneumoniae*, 1 *S. mitis*, and 1 unidentified isolate. The 110 non-invasive disease isolates were identified as 53 *S. pseudopneumoniae*, 22 *S. pneumoniae*, 12 *S. mitis*, and 23 unidentified isolates. In all groups sporadic alleles associated in the MLST database with typical pneumococci were noted (Table S1). The phenotypic and genotypic characteristics of each group of isolates are summarized in Table 1 and are discussed below.

**Table 1:** Phenotypic and genotypic characterization of MLSA typeable isolates.

	MLSA classification (%)			
	*S. pseudopneumoniae* (n = 61)	*S. pneumonia*		*S. mitis* (n = 13)
		typeable (n = 23)	nontypeable (n = 11)	
**Phenotypic characterization**				
optochin susceptibility (≥14 mm)				
5% CO_2_	10 (16.4)	8 (34.8)	6 (54.6)	4 (30.8)
ambient atmosphere	39^a^ (63.9)	21 (91.3)	6^b^ (54.6)	8^c^ (61.5)
bile solubility	22 (36.1)	22 (95.7)	11 (100)	2 (15.4)
**Genotypic characterization**				
PCR-based				
pneumococcal *lyt*A	0 (0)	23 (100)	11 (100)	0 (0)
pneumococcal specific 16S-rRNA	49 (80.3)	23 (100)	11 (100)	2 (15.4)
*Spn*9802	59 (96.7)	23 (100)	8 (72.7)	8 (61.5)
*psp*A	1 (1.6)	21 (91.3)	10 (90.9)	7 (53.8)
*cps*A	0 (0.0)	17 (73.9)	2 (18.2)	0 (0)
*ali*B-like ORF2	61 (100.0)	7 (30.4)	9 (81.8)	12 (92.3)
RFLP signatures				
pneumococcal *lyt*A/atypical *lytA*	0 (0)/61 (100)	23 (100)	9 (81.8)/2 (18.2)	0/11(84.6)^d^
*ply/mly*	7 (11.5)/54 (88.5)	23 (100)/0 (0)	11 (100)/0	2 (15.4)/7 (53.8)^e^

a 11 strains did not grow in an ambient atmosphere, among the 39 isolates susceptible to optochin in ambient atmosphere, 31 were resistant in CO_2_.

b 3 strains did not grow in ambient atmosphere.

c 2 strains did not grow in ambient atmosphere.

d 2 strains were not screened.

e 2 strains did not amplify, 2 yielded a mixed pattern.

### *S. pseudopneumoniae*

A total of 61 *S. pseudopneumoniae* were identified by MLSA and were further analyzed. The clinical sources of the *S. pseudopneumoniae* isolates were: sputum (n = 32), bronchial aspirate (n = 17), bronchoalveolar lavage (n = 4), blood (n = 2), conjunctiva (n = 2), nasal swab (n = 1), bronchoscopic-protected catheter brush (n = 1), pharyngeal swab (n = 1), and ascitic fluid (n = 1). The majority (88.5%) of the *S. pseudopneumoniae* were isolated from adults, and the male gender was predominant (68.9%) (data not shown).

Antimicrobial non-susceptibility rates were high against penicillin (60.7%) and erythromycin (42.6%), as shown in Table 2. Among the 26 macrolide-resistant isolates, the MLSB phenotype and the M phenotype were equally distributed. Only nine *S. pseudopneumoniae* isolates were fully susceptible to all antimicrobials tested. High macrolide-resistance rates have been described among isolates recovered from respiratory samples from New Zealand, [40] and France. [18] Fluoroquinolone resistant isolates have also been described. [41] The high antimicrobial resistance rates together with the confirmation of the ability of this microorganism to cause invasive diseases raises this pathogen as a real clinical concern.

**Table 2:** Antimicrobial susceptibility of 61 *S. pseudopneumoniae* clinical isolates.

Antibiotic	MIC (mg/L)			No. non-susceptible isolates (%)
	Range	MIC_50_	MIC_90_	
Penicillin	≤0.03–2	≤0.03	0.5	37 (60.7%)
Cefotaxime	≤0.03–1	≤0.12	0.25	2 (3.3%)
Erythromycin	≤0.12–≥128	≤0.12	≥32	26 (42.6%)
Clindamycin	≤0.12–≥128	≤0.12	≥0.5	13 (21.3%)
Cotrimoxazole	≤0.5/9.5–≥2/38	≤0.5/9.5	≥2/38	24 (39.3%)
Tetracycline	≤0.12–64	≤0.25	4	18 (29.5%)
Ciprofloxacin	≤0.12–32	≤1	≤1	6 (9.8%)
Levofloxacin	≤0.12–≥16	≤1	≤1	3 (4.9%)
Chloramphenicol	≤2–4	≤2	≤2	0 (0%)

The 61 *S. pseudopneumoniae* isolates displayed heterogeneous profiles regarding several of the phenotypic and genotypic characterization assays that were performed (Table 1). In particular, 16.4% of the isolates were susceptible to optochin in a 5%CO_2_-enriched atmosphere and 63.9% were susceptible in ambient atmosphere. Only 50.8% of the *S. pseudopneumoniae* isolates displayed the typical phenotype originally described for this species (optochin-resistant in CO_2_ but susceptible in O_2_ atmosphere). Also, 36.1% of the isolates were bile soluble. Although these biochemical traditional identification tests are the first step for phenotypic identification of *S. pseudopneumoniae*, in the present study we observed that these characteristics were frequently diverse among the isolates of this species, as previously shown. [42].

Screening for genetic markers described by others as species-specific for *S. pneumoniae* – specific 16S-rRNA, *Spn9802, pspA* and *ply* - revealed their presence in some *S. pseudopneumoniae* isolates in contrast with previous publications. [22], [23], [43] No *S. pseudopneumoniae* isolates harbored the pneumococcal *lytA* nor the *cps*A capsular gene. The *ali*B-like ORF2 was present in all isolates. The lack of *cpsA* was in line with previous observations that suggest *S. pseudopneumoniae* lacks a pneumococcus-like capsule. [44].

**Figure 2:**
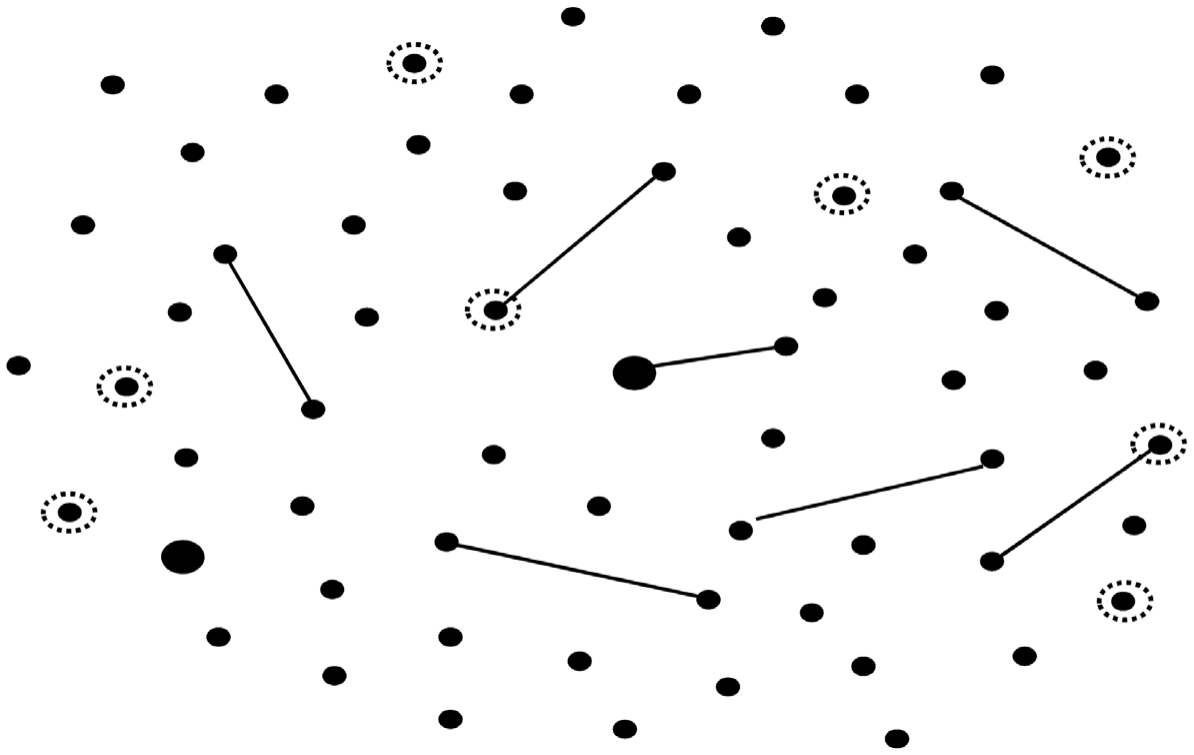
Representation of the *S. pseudopneumoniae* population by eBURST analysis. Each point represents a different allele combination. Solid lines, single-locus variants; dashed circles, invasive disease isolates; larger circles indicate two isolates with the same allele combination.

A high clonal diversity was found as 59 allelic profiles were detected by MLST (Figure 2 and Table S1). By e-BURST seven clonal groups were identified and each contained only two allelic profiles. On two occasions, pairs of isolates were found to have the same allelic profile. No association between isolates sharing a same allelic profile or being in the same clonal group was obvious.

### *S. pneumoniae*

Out of 34 *S. pneumoniae* identified in this collection, 23 isolates previously identified as non-(sero)typeable pneumococci were in fact capsulated when reanalyzed; the other 11 were confirmed as non-typeable. Several explanations could be put forward to justify why isolates previously identified as atypical pneumococcal were found to be capsulated upon reanalysis. For example, differences in the quality of the antisera, lack of capsular production due to passage of isolates on agar plates, and human error.

The clinical sources of the capsulated isolates were sputum (n = 14), bronchoalveolar lavage (n = 2), blood (n = 5), transthoracic needle aspiration (n = 1), and umbilical swab (n = 1). The majority were isolated from adults (87.5%), and the male gender was predominant (75.0%). The clinical sources of the non-capsulated isolates were blood (n = 4), sputum (n = 4), conjunctival swab (n = 2), and nasal swab (n = 1). The majority (90%) were isolated from adults, and 40% were males (data not shown).

Among the capsulated isolates, the most frequent serotypes were 38 and 6B (3 isolates each, Table 3). Interestingly, these serotypes were also frequently misidentified as atypical pneumococci in a recent study from the USA. [45] This observation may indicate that some representatives of these serotypes may be hard to visualize by the Quellung reaction, leading to misidentification, or that these serotypes may contain unknown different subtypes.

**Table 3:** Properties of *S. pneumoniae* clinical isolates.

Serotypes	Sequence type^a^(no. of isolates)	MLST allelic profile	Antimicrobial non-susceptibility pattern^b^	Observations^c^
6B	90 (1)	5-6-1-2-6-3-4	PEN, TET, ERY, CLI, CTX	Spain^6B^-ST90
	94 (1)	5-6-1-2-6-3-54	PEN, TET, CHL, ERY, CLI, SXT, CIP	Spain^6B^-ST90 SLV
	**8270 (1)**	32-28-1-1-15-52-15	TET, ERY, CLI	
38	393 (2)	10-43-41-18-13-49-6	Susceptible	
	**8278 (1)**	10-61-41-18-13-49-6	Susceptible	
13	70 (1)	2-13-1-4-6-12-1	Susceptible	
	**8271 (1)**	7-13-**368**-4-6-1-20	Susceptible	
19F	89 (1)	5-5-7-7-8-5-1	PEN, TET, CHL, SXT	
	**8275 (1)**	5-5-7-7-8-5-**538**	PEN, TET, CHL, ERY, CLI, CTX, SXT	
25A	393 (1)	10-43-41-18-13-49-6	Susceptible	
	**8274 (1)**	10-43-41-18-13-37-6	PEN, SXT	
3	180 (1)	7-15-2-10-6-1-22	Susceptible	Netherlands^3^-ST180
4	247 (1)	16-13-4-5-6-10-14	Susceptible	
7F	2178 (1)	10-20-14-1-6-20-29	TET	Denmark^12F^-ST218 SLV
10A	**8272 (1)**	5-13-4-4-6-1-20	Susceptible	
17A	**8277 (1)**	5-**365**-2-16-6-3-245	Susceptible	
18C	191 (1)	8-9-2-1-6-1-17	Susceptible	Netherlands^7F^-ST191
19A	81 (1)	4-4-2-4-4-1-1	PEN, TET, CHL, ERY, CLI, CTX, SXT, CIP, LEV	Spain^23F^-ST81
20	**8269 (1)**	15-**364**-8-18-15-1-31	Susceptible	
22F	2104 (1)	2-16-1-4-6-1-1	Susceptible	
33F	1012 (1)	2-5-29-18-42-3-18	TET, ERY, CLI	
35A	1273 (1)	10-12-4-12-9-28-18	Susceptible	
NT	448 (2)	8-5-2-27-2-11-71	Susceptible	USA^NT^-ST448
	508 (2)	13-8-65-1-60-16-6	Susceptible	
	66 (1)	2-8-2-4-6-1-1	PEN, TET, SXT, CIP, LEV	
	72 (1)	2-13-2-4-9-4-1	Susceptible	
	344 (1)	8-37-9-29-2-12-53	PEN, TET, ERY, SXT	Norway^NT^-ST344
	942 (1)	8-10-15-27-2-28-4	PEN, SXT	
	**8268 (1)**	8-10-84-1-2-14-4	Susceptible	
	**8273 (1)**	8-37-2-27-2-11-53	Susceptible	USA^NT^-ST448 DLV
	**8276 (1)**	8-178-9-29-2-12-15	PEN, TET, ERY, CLI, SXT	Norway^NT^-ST344 DLV

a Novel STs and alleles found in this study are represented in bold.

b PEN, penicillin; CTX, cefotaxime; ERY, erythromycin; CLI, clindamycin; TET, tetracycline; CHL, chloramphenicol, SXT, trimethoprim-sulfamethoxazole non-susceptible; CIP, ciprofloxacin; LEV, levofloxacin.

c International clones of PMEN; SLV, Single Locus Variant; DLV, Double Locus Variant.

Multiresistance (non-susceptibility to three or more classes of antimicrobials) was found among 11 isolates (3 were from invasive disease) and was associated to NT (n = 4), and serotypes 6B (n = 3), 19F (n = 2), 19A (n = 1), and 33F (n = 1) (Table 3). Three of the eleven NT isolates were multiresistant. A high frequency of multiresistance among non-typeable strains has been observed in other studies. [7], [9].

Regarding the classical presumptive identification of pneumococci based on optochin susceptibility in CO_2_ atmosphere and bile solubility, many exceptions were found among this group of isolates: 20 were optochin resistant and one was bile insoluble. Although rare, these exceptional phenotypes were previously reported in other studies. [46].

Genotypic analysis showed the ubiquitous presence of pneumococcal *lytA*, specific16S-rRNA, and *ply*. *Spn9802* was present in all but three non-typeable isolates contrasting with previous publications that suggested that this ORF was ubiquitous in pneumococcus. [22], [23].

The *lytA*-typical pneumococcal RFLP signature was identified in all but two isolates. The two exceptions were associated with a novel signature also distinct from the characteristic atypical pattern associated with non-pneumococcal isolates. The molecular basis of this novel signature is currently being investigated.

The capsular gene *cps*A was present in most capsulated isolates with the exception of those of serotypes 25A and 38 in agreement with published literature. [9], [47] Instead, isolates of serotype 25A and 38 had *aliB*-likeORF2 as described, [9] which was also detected in single isolates of serotype 35A. Among non-typeable isolates, nine had aliB-like ORF2 and two had *cpsA.* A possible explanation for this latter observation is that the isolates may have lost the capacity to produce a capsule *in vitro* [14] due to alterations in the capsular genes. [48].

MLST analysis of the *S. pneumoniae* isolates showed that close to one-third (32.4%) had novel allelic profiles. Of interest, six of the nine allelic profiles identified among the non-typeable pneumococcal isolates were previously identified in other countries and were also associated to non-serotypeability. The international PMEN lineages USA^NT^-ST448 and Norway^NT^-ST344 accounted for five isolates, three having been recovered from invasive disease. Non-typeable pneumococci were previously found not only among colonization, but also as causative agents of acute otitis media and conjunctivitis. [7], [9], [11], [12], [14] The association of MLST lineages exclusive of non-capsulated isolates to invasive disease has only been described recently. [14] These observations suggest that, in spite of their sporadic occurrence, non-typeable pneumococci have a higher clinical impact than previously thought as they have been associated with a varied spectrum of infections including invasive disease.

### *S. mitis*

Although the 13 *S. mitis* isolates were phenotypically and genotypically heterogeneous, *lytA* analyses (in addition to MLSA) consistently suggested they were not pneumococci. Of interest, and as observed for some *S. pseudopneumoniae* isolates, a few of the *S. mitis* harboured genetic markers – *Spn9802*, *pspA* and *ply* - previously associated to pneumococci. The occurrence of *S. mitis* isolates harbouring genes encoding *S. pneumoniae* virulence factors has been described, [15], [26] and led to the suggestion that identification of this group of bacteria by a single identification marker may not be possible as horizontal gene transfer between them can occur. [24], [27].

Regarding antimicrobial susceptibility, 84.6% were non-susceptible to penicilin and 69.2% were multidrug resistant. Most of the isolates (12/13) were recovered from non-invasive disease; however, one isolate was recovered from bronchoalveolar lavage. *S. mitis* isolates have been previously associated with disease, [36], [49], [50] and high levels of antimicrobial resistance. [15], [51].

### Non-classified Isolates

Close to one-fifth of the isolates (18.2%) remained non-classified. Although MLSA associated to the MLST *S. pneumoniae* scheme works well to identify atypical isolates, we were unable to apply it to 24 isolates due to lack of amplification of some DNA fragments with the combinations of primers that are routinely used for *S. pneumoniae*. For these isolates, alternative primers, MLSA schemes or assays would have been needed. [52] Of note, only one isolate was recovered from invasive disease.

### Conclusions

In summary, among disease isolates classified as atypical pneumococci, close to half (46.2%) were *S. pseudopneumoniae*, and only a quarter were pneumococci (17.4% were capsulated and 8.3% were non-typeable). In addition, 9.8% were *S. mitis* and the rest were non-pneumococci that remained unidentified. In agreement with other studies, we found that many of the currently proposed methodologies to distinguish pneumococci from closely-related species are not species-specific. Furthermore, *S. pseudopneumoniae* that failed to have the optochin phenotypes described by Arbique et al. were also identified.

We found that *S. pseudopneumoniae* have low clonality and that antimicrobial resistance is well-disseminated is this species. Our study stresses the clinical role of *S. pseudopneumoniae* and non-typeable pneumococci since they have the capacity to cause invasive disease and the high antimicrobial resistance rates are of concern.

## Supporting Information

Table S1 **MLST allelic profiles of non-pneumococcal isolates.** Invasive strains are indicated in bold. Most alleles are divergent from all the alleles described at the *S. pneumoniae* MLST database as of July 26, 2012. The allele number of the closest match is indicated; similarity (in %) is indicated in parenthesis. ND, not determined.
(DOCX)
