# Supplementary material for: Disease Isolates of Streptococcus pseudopneumoniae and Non-Typeable S. pneumoniae Presumptively Identified as Atypical S. pneumoniae in Spain
Source: PLoS One. 2013 Feb 21;8(2):e57047. doi: 10.1371/journal.pone.0057047 (PMC3578818; doi:10.1371/journal.pone.0057047)
Supplement: Table S1 — MLST allelic profiles of non-pneumococcal isolates. Invasive strains are indicated in bold. Most alleles are divergent from all the alleles described at the S. pneumoniae MLST database as of July 26, 2012. The allele number of the closest match is indicated; similarity (in %) is indicated in parenthesis. ND, not determined. (DOCX) [file pone.0057047.s001.docx]

**TABLE S1**

| **Strain ref.** | **MLSA classification** | **MLST allelic profile** | | | | | | |
| --- | --- | --- | --- | --- | --- | --- | --- | --- |
|  |  | ***aroE*** | ***gdh*** | ***gki*** | ***recP*** | ***spi*** | ***xpt*** | ***ddl*** |
| 37 | *S. pseudopneumoniae* | 103 (99) | 166 | 94 (99) | 54 (96) | 56 (96) | 144 (98) | ND |
| **281** | *S. pseudopneumoniae* | 103 (99) | 166 (99) | 94 (99) | 54 (96) | 56 (96) | 144 (98) | 114 (97) |
| 337 | *S. pseudopneumoniae* | 103 (99) | 94 (98) | 89 | 135 (96) | 56 (96) | 46 (95) | ND |
| 531 | *S. pseudopneumoniae* | 139 | 91 (98) | 40 (97) | 41 (96) | 67 (95) | 153 (96) | ND |
| 603 | *S. pseudopneumoniae* | 139 | 94 (98) | 94 (99) | 67 (96) | 56 (96) | 22 (98) | ND |
| 713 | *S. pseudopneumoniae* | 103 (97) | 94 (97) | 2 | 139 (97) | 251 (97) | 151 (96) | 348 (97) |
| 939 | *S. pseudopneumoniae* | 139 (99) | 94 (97) | 66 (96) | 139 (98) | 251 (97) | 105 (98) | ND |
| 1137 | *S. pseudopneumoniae* | 103 | 166 | 29 | 37 (99) | 6 | 151 (96) | ND |
| 1173 | *S. pseudopneumoniae* | 139 | 94 (98) | 1 (99) | 139 (96) | 56 (96) | 307 (98) | ND |
| 1304 | *S. pseudopneumoniae* | 139 | 165 (98) | 94 (99) | 75 (97) | 251 (97) | 51 (96) | 85 (96) |
| 1324 | *S. pseudopneumoniae* | 103 | 166 (99) | 40 (97) | 67 (96) | 6 | 72 (97) | ND |
| 1544 | *S. pseudopneumoniae* | 139 | 40 (99) | 107 (96) | 135 (96) | 64 (96) | 151 (96) | ND |
| 1752 | *S. pseudopneumoniae* | 139 | 57 (96) | 1 | 67 (97) | 82 (96) | 51 (96) | ND |
| 1848 | *S. pseudopneumoniae* | 103 (99) | 166 (97) | 94 (97) | 107 (97) | 56 (96) | 51 (96) | ND |
| 1850 | *S. pseudopneumoniae* | 103 (99) | 40 (99) | 185 (97) | 135 (96) | 130 (96) | 51 (96) | ND |
| 1927 | *S. pseudopneumoniae* | 103 (99) | 166 (98) | 66 (96) | 139 (97) | 130 (96) | 307 (96) | ND |
| 2161 | *S. pseudopneumoniae* | 139 | 166 (98) | 66 (96) | 59 (99) | 251 (97) | 151 (96) | ND |
| 2353 | *S. pseudopneumoniae* | 103 (99) | 166 | 53 | 135 (96) | 56 (96) | 151 (96) | ND |
| 2504 | *S. pseudopneumoniae* | 103 (99) | 132 (97) | 4 | 139 (96) | 56 (96) | 307 (98) | ND |
| 2522 | *S. pseudopneumoniae* | 139 | 165 (98) | 94 (99) | 135 (96) | 214 (96) | 51 (96) | ND |
| 2565 | *S. pseudopneumoniae* | 103 (99) | 166 (97) | 66 (96) | 67 (96) | 56 (96) | 228 | ND |
| 2581 | *S. pseudopneumoniae* | 139 | 94 (98) | 94 (99) | 67 (96) | 329 (96) | 307 (98) | ND |
| 2597 | *S. pseudopneumoniae* | 103 (99) | 40 (99) | 1 (99) | 135 (96) | 64 (96) | 151 (96) | ND |
| 2609 | *S. pseudopneumoniae* | 139 (99) | 57 (99) | 66 (96) | 135 (96) | 56 (96) | 51 (96) | ND |
| **2615** | *S. pseudopneumoniae* | 139 | 166 (97) | 94 (97) | 107 (97) | 56 (96) | 51 (96) | 360 (98) |
| 2621 | *S. pseudopneumoniae* | 139 | 166 | 40 (97) | 37 (99) | 6 | 1 | 85 (96) |
| 3075 | *S. pseudopneumoniae* | 103 (99) | 40 (99) | 66 (96) | 135 (96) | 56 (96) | 151 (96) | ND |
| 3194 | *S. pseudopneumoniae* | 139 | 166 (99) | 40 (97) | 37 (99) | 6 | 307 (98) | ND |
| 3205 | *S. pseudopneumoniae* | 139 | 40 (98) | 72 (99) | 54 (96) | 56 (96) | 307 (98) | ND |
| **3473** | *S. pseudopneumoniae* | 103 (99) | 40 | 182 (97) | 29 | 56 (96) | 105 (96) | 65 (94) |
| 3738 | *S. pseudopneumoniae* | 139 | 94 (98) | 44 (98) | 139 (97) | 56 (96) | 307 (98) | ND |
| **4526** | *S. pseudopneumoniae* | 103 (99) | 40 (99) | 345 | 135 (96) | 64 (96) | 151 (96) | ND |
| 6265 | *S. pseudopneumoniae* | 103 (99) | 57 (98) | 348 (96) | 67 (96) | 251 (97) | 228 | 85 (96) |
| 6338 | *S. pseudopneumoniae* | 103 (99) | 40 (99) | 51 (97) | 135 (98) | 251 (96) | 307 (98) | 78 (97) |
| 6339 | *S. pseudopneumoniae* | 103 (99) | 40 (99) | 51 (97) | 135 (98) | 251 (96) | 307 (98) | ND |
| 6408 | *S. pseudopneumoniae* | 103 (99) | 290 (97) | 94 (99) | 54 (98) | 251 (97) | 51 (96) | 114 (97) |
| 6486 | *S. pseudopneumoniae* | 11 (98) | 321 (97) | 345 | 54 (97) | 251 (97) | 51 (96) | 360 (98) |
| 6669 | *S. pseudopneumoniae* | 139 | 40 (98) | 350 (99) | 1 | 251 (97) | 153 (96) | ND |
| 6744 | *S. pseudopneumoniae* | 139 | 40 (98) | 40 (97) | 29 | 251 (97) | 51 (96) | ND |
| 6787 | *S. pseudopneumoniae* | 139 | 52 (98) | 345 | 4 | 251 (97) | 47 (99) | ND |
| 6905 | *S. pseudopneumoniae* | 139 | 94 (98) | 29 | 67 (98) | 251 (97) | 22 (98) | 18 |
| 7253 | *S. pseudopneumoniae* | 139 | 40 (98) | 345 | 67 (96) | 251 (97) | 307 (98) | 6 |
| 7327 | *S. pseudopneumoniae* | 139 | 166 (98) | 345 (99) | 59 (99) | 251 (97) | 153 (96) | ND |
| 7332 | *S. pseudopneumoniae* | 103 | 40 (99) | 345 | 135 (96) | 64 (96) | 151 (96) | ND |
| 7842 | *S. pseudopneumoniae* | 103 (99) | 94 (98) | 94 (96) | 139 (97) | 6 (99) | 144 (98) | ND |
| **7943** | *S. pseudopneumoniae* | 103 (99) | 94 (98) | 66 (96) | 59 (99) | 56 (96) | 51 (96) | 360 (98) |
| **8615** | *S. pseudopneumoniae* | 139 | 94 (98) | 9 (98) | 1 | 56 (96) | 51 (96) | 300 |
| 8646 | *S. pseudopneumoniae* | 103 (99) | 166 (98) | 86 (96) | 139 (97) | 251 (97) | 153 (96) | ND |
| 8937 | *S. pseudopneumoniae* | 103 (99) | 166 (97) | 94 (97) | 54 (97) | 56 (96) | 51 (96) | ND |
| 8971 | *S. pseudopneumoniae* | 103 (99) | 145 (97) | 94 (97) | 139 (97) | 214 (96) | 153 (96) | ND |
| 9012 | *S. pseudopneumoniae* | 139 | 40 (99) | 345 (97) | 135 (96) | 251 (97) | 105 (98) | 300 |
| 9013 | *S. pseudopneumoniae* | 139 | 94 (98) | 94 (99) | 67 (96) | 82 (95) | 307 (98) | ND |
| **9015** | *S. pseudopneumoniae* | 139 | 94 (98) | 84 (98) | 67 (96) | 159 (94) | 307 (98) | 85 (96) |
| 9096 | *S. pseudopneumoniae* | 103 (99) | 166 (98) | 345 | 107 (97) | 251 (97) | 51 (96) | 85 (96) |
| **9111** | *S. pseudopneumoniae* | 103 (99) | 166 (97) | 94 (97) | 107 (94) | 56 (96) | 51 (96) | 81 (97) |
| 9230 | *S. pseudopneumoniae* | 139 (99) | 40 (98) | 345 (98) | 54 (97) | 56 (96) | 307 (98) | ND |
| 9275 | *S. pseudopneumoniae* | 139 | 165 (98) | 40 (97) | 135 (96) | 251 (97) | 51 (96) | 85 (96) |
| 9545 | *S. pseudopneumoniae* | 103 | 165 (98) | 250 (95) | 75 (97) | 6 | 307 (98) | ND |
| 9731 | *S. pseudopneumoniae* | 103 (99) | 166 (98) | 49 (95) | 2 | 315 (95) | 51 (96) | ND |
| 9781 | *S. pseudopneumoniae* | 139 | 166 (98) | 49 (95) | 59 (99) | 315 (95) | 8 | ND |
| 9786 | *S. pseudopneumoniae* | 103 (99) | 165 (98) | 345 | 139 (97) | 6 | 51 (95) | ND |
| 1964 | *S. mitis* group | 165 (96) | 104 (99) | 50 (95) | 221 (96) | 56 (96) | 221 (96) | ND |
| 6147 | *S. mitis* group | 143 (92) | 40 (99) | 335 (96) | 133 (92) | 64 (96) | 314 | 179 (94) |
| 6760 | *S. mitis* group | 29 (95) | 290 (98) | 49 (95) | 50 (98) | 273 (97) | 153 (96) | ND |
| 7728 | *S. mitis* group | 242 (99) | 172 (98) | 48 (97) | 38 (97) | 273 (95) | 46 | ND |
| 7755 | *S. mitis* group | 242 (99) | 172 (97) | 48 (97) | 67 (97) | 99 (95) | 46 | ND |
| 8271 | *S. mitis* group | 88 (96) | 132 (98) | 87 (97) | 107 (95) | 193 (95) | 153 (96) | ND |
| 8277 | *S. mitis* group | 59 (93) | 182 (97) | 48 (97) | 10 (97) | 214 (97) | 151 (95) | ND |
| 8447 | *S. mitis* group | 169 (96) | 174 (97) | 48 (97) | 50 (98) | 67 (95) | 293 (96) | ND |
| 8592 | *S. mitis* group | 106 (95) | 52 (97) | 250 (95) | 67 (97) | 159 (94) | 308 (95) | ND |
| 8692 | *S. mitis* group | 106 (95) | 52 (97) | 250 (95) | 67 (96) | 159 (94) | 308 (95) | ND |
| **8943** | *S. mitis* group | 143 (95) | 57 (98) | 2 (98) | 135 (96) | 85 (97) | 144 (95) | 102 (98) |
| 9279 | *S. mitis* group | 169 (96) | 172 (97) | 48 (97) | 75 (97) | 273 (95) | 46 (99) | 101 (97) |
| 9597 | *S. mitis* group | 165 (95) | 290 (97) | 335 (91) | 40 (97) | 265 (95) | 153 (96) | ND |
| 8482 | Undefined | 5 | 145 (97) | 18 (96) | 135 (98) | 68 (96) | 293 (96) | 232 (97) |
| 7693 | Undefined | 15 | 103 (97) | 351 (97) | 54 (97) | 55 (95) | 40 | 115 (94) |
